# Synthesis, Characterization, Spectral Studies and Antifungal Activity of Mn(II), Fe(II), Co(II), Ni(II), Cu(II) and Zn(II) Complexes with 2-(4- Sulphophenylazo)-1,8-Dihydroxy-3,6-Napthalene Disulphonic Acid Trisodium Salt

**DOI:** 10.1155/BCA.2005.217

**Published:** 2005

**Authors:** Gajanan Pandey, K. K. Narang

**Affiliations:** Department of Applied Chemistry Institute of Technology Banaras Hindu University Varanasi India

## Abstract

Complexes of the type Na_6_[M(HL)_2_(H_2_O)_2_], where M= Mn(II), Fe(II), Co(II), Ni(II), Cu(II) and Zn(II) and Na_3_H_2_L= 2-(4-sulphophenylazo)-1,8-dihydroxy 3,6 naphthalene disulphonic acid trisodium salt, have been synthesized and characterized by physico-chemical (elemental analyses, solubility, electrolytic conductance, magnetic susceptibility measurement) and spectral (UV-Visible, IR, ESR, powder x-ray diffraction) techniques for their structure and studied for their antifungal activity against ten fungi. The anionic 1:2 metal:ligand complexes show octahedral geometry around M(II), a significant antifungal activity against *Curvularia lunata* and *Alternaria triticina* and a moderate activity against *Alternaria brassicicola*, *Alternaria brassicae*, *Alternaria solanae*, *Curvularia species*, *Helminthosporium oryzae*, *Collectotrichum capsici*, *Aspergillus niger*, *Aspergillus flavus* and *Fusarium udum*.


### Synthesis, Characterization, Spectral Studies and Antifungal Activity of Mn(II), Fe(II), Co(II), Ni(II), Cu(II) and Zn(II) Complexes with 2-(4-Sulphophenylazo)-1,8-Dihydroxy-3,6-Napthalene Disulphonic Acid Trisodium Salt

Gajanan Pandey* and K.K. Narang

*Department of Applied Chemistry*
*Institute of Technology, Banaras Hindu University, Varanasi, India*
*E-mail : pandeygajanan@rediffmail.com*

### ABSTRACT

Complexes of the type $Na_6[M(HL)_2(H_2O)_2]$, where M= Mn(II), Fe(II), Co(II), Ni(II), Cu(II) and Zn(II) and $Na_3H_2L$= 2-(4-sulphophenylazo)-1,8-dihydroxy 3,6 naphthalene disulphonic acid trisodium salt, have been synthesized and characterized by physico-chemical (elemental analyses, solubility, electrolytic conductance, magnetic susceptibility measurement) and spectral (UV-Visible, IR, ESR, powder x-ray diffraction) techniques for their structure and studied for their antifungal activity against ten fungi. The anionic 1:2 metal:ligand complexes show octahedral geometry around M(II), a significant antifungal activity against *Curvularia lunata* and *Alternaria triticina*and and a moderate activity against *Alternaria brassicicola, Alternaria brassicae, Alternaria solanae, Curvularia species, Helminthosporium* oryzae, *Collectotrichum capsici, Aspergillus niger, Aspergillus flavus* and *Fusarium udum.*

### INTRODUCTION

Several azo compounds containing, -N=N- group have been used in colorimetric /1/, chromogenic /2/, spectrophotometric /3/, complexometric /4/ studies as well as indicators in titrimetric determination of metal ions /5/. α-Hydroxy azo compounds are significant due to their use as models for metal-enzyme interactions and transport of metal ions in biological fluids /6/. Polydentate chelating agents are able to remove metal ions from metalloenzymes by forming ternary complexes /7/. Coordinating abilities of aromatic α-hydroxy azo dyes namely, 1, 4-bis-p-sulphonylazo-2,3-dihyroxynaphthalene, 4-(2-hydroxy-1-phenylazo)-benzensulphonate, 4-(2-hydroxy-1-naphthylazo)-benzenesulphonate and 4-(9-hydroxy-10-phenanthrylazo)-benzenesulphonate, towards Cu(II), Co(II), Ni(II) and Zn(II) show both mono and binuclear chelates formation /8/. 2-(4-sulphophenylazo)-1,8-dihydroxy 3,6 naphthalene disulphonic acid trisodium salt, $Na_3H_2L$

is known to be weak ligand but still forms metal chelates in solution /9/.

Coordination compounds have also been studied as antitumour /10/ antiviral /11/ and antimalerial /12/ agents. The ability of metal ions to form complexes with ligands containing nitrogen, oxygen and sulphur donor atoms /13/ is related to such bioactivity. Schiff base complexes have led to a better understanding of the factors modifying the coordination sphere and the electronic properties of metals ions, and of chelate formation. Hydroxy azo compounds are structurally related to the Schiff bases, with an added advantage of being soluble and stable in water allowing a closure understanding of biological processes. X-ray crystal structure analysis of azo phenolate derivatives and their metal complexes has been done for bioinorganic purposes /14, 15/. Complexes of amino acids with sulphonated azo dyes have been used as model systems for understanding the biomolecular recognition of glycosamino glycans for proteins. Further, sulphonato azo dyes, Evans blue and Congo Red are being studied as HIV inhibitors of viral replications. The latter effect is believed to be caused by binding of azo dyes to both protease and reverse transcriptase of this virus /16/.

Metal complexes have been proved to be more fungitoxic compared to the basic organic compound /17/. Dithocarbamate, morpholine dithiocarbamates and diphenyl dithiocarbamates complexes of Zn(II) Mn(II), Fe(II) and Cu(II) have been found to be very active against certain fungi, namely *Helminthosporium goffybii* and *Alternaria solanae*. Copper(II) sulphate and copper(II) oxychlorides are very effective against species of *Aspergillus*, *Penicillium*, *Fusarium* and *Gliocledium*, somewhat more on *Alternaria*, *Chaetomium* and *Trichothesium* /18/. Further, it has been demonstrated that chelation in these compounds, to a large extent, is responsible for antifungal and antibacterial activity /19/. Transition metal complexes with dyes e.g. Cu(II) with methyl Orange have also shown pronounced antifungal and antibacterial activity /20/. Copper complexes of thiophene oligomers have proved to be excellent fungicidal and also possessing other antimicrobial property against *Aspergillus niger*. These effects were attributed to a slow release of $Cu^{2+}$ into the culture medium, thereby causing inhibition of fungus /21/.

In view of the above studies, our interests have grown in this field. The objective of the present study, therefore was to synthesize and characterize Mn(II), Fe(II), Co(II), Ni(II), Cu(II) and Zn(II) complexes with title ligand, $Na_3H_2L$ and to screen these metal complexes against several fungi by studying their antifungal properties.

### EXPERIMENTAL

### Materials

Metal salts used in the present study were of BDH AR or equivalent grade. Ethanol, methanol, dioxane etc. were purified by distillation. 2-(4-sulphophenylazo)-1,8-dihydroxy 3,6 naphthalene disulphonic acid tri sodium salt $Na_3H_2L$, was purified by literature method /22/.

### Synthesis of Complexes

To an aqueous solution of the ligand, 2(4-sulphophenylazo)-1,8,dihydroxy-3,6-napthalene disulphonic acid trisodium salt, $Na_3H_2L$ (ca 2 m mol, 1.14082 g), aqueous solutions of metal salts, (ca 1 m mol, 0.197 g of $MnCl_2 \cdot 4H_2O$, 0.2780 g of $FeSO_4 \cdot 7H_2O$, 0.281 g of $CoSO_4 \cdot 7H_2O$, 0.2809 g of $NiSO_4 \cdot 7H_2O$, 0.2497 g of $CuSO_4 \cdot 5H_2O$ and 0.2870 g of $ZnSO_4 \cdot 7H_2O$) were respectively added with constant stirring. These solution mixtures were then digested on water bath with constant stirring at about 95 °C. The solutions were made alkaline (~ 8-9 pH) by adding NaOH solutions and further refluxed for about 2 hours. The solutions were concentrated up to half of the volume by heating on water bath and then about 10 ml absolute ethanol/methanol was added. The reaction mixtures were further refluxed for ca two hours. Shinning microcrystals, thus obtained, were filtered, washed with ethanol, several times, and dried over $P_2O_5$ under reduced pressure. Recrystalization was carried out from ethanol solution. Their and physico-chemical and elemental data are presented in Table-1.

**Table 1**

Analytical Data, Magnetic Moment and Physical Properties of Complexes of $Na_3H_2L$ with Mn(II), Fe(II), Co(II), Ni(II), Cu(II) and Zn(II)

| Complexes and Molecular wt. | Colour | % Found (calculated) | | | | | | Effective magnetic moment (BM) | $\Lambda_m$, $\Omega^{-1}$, $cm^2 mol^{-1}$ (water) |
|---|---|---|---|---|---|---|---|---|---|
| | | Na | M | C | H | N | S | | |
| $Na_6[Mn(HL)_2(H_2O)_2]$ | Reddish brown | 11.17 (11.22) | 4.42 (4.47) | 31.20 (31.22) | 1.63 (1.63) | 4.56 (4.55) | 15.61 (15.62) | 6.12 | 702 |
| $Na_6[Fe(HL)_2(H_2O)_2]$ | Black | 11.19 (11.21) | 4.50 (4.54) | 31.21 (31.20) | 1.61 (1.62) | 4.53 (4.55) | 15.61 (15.60) | 4.97 | 701 |
| $Na_6[Co(HL)_2(H_2O)_2]$ | Blood red | 11.16 (11.18) | 4.77 (4.78) | 31.11 (31.12) | 1.61 (1.62) | 4.53 (4.54) | 15.55 (15.56) | 4.59 | 698 |
| $Na_6[Ni(HL)_2(H_2O)_2]$ | Reddish brown | 11.20 (11.19) | 4.76 (4.75) | 31.03 (31.13) | 1.60 (1.62) | 4.53 (4.54) | 15.51 (15.56) | 2.9 | 708 |
| $Na_6[Cu(HL)_2(H_2O)_2]$ | Violet | 11.15 (11.14) | 5.17 (5.13) | 31.20 (31.00) | 1.60 (1.61) | 4.51 (4.52) | 15.53 (15.50) | 1.9 | 715 |
| $Na_6[Zn(HL)_2(H_2O)_2]$ | Reddish brown | 11.11 (11.13) | 5.21 (5.27) | 30.91 (30.96) | 1.61 (1.61) | 4.50 (4.51) | 15.46 (15.48) | - | 711 |

HL = $C_{16}H_8N_2O_{11}S_3$; Yields are about 80 % in all the cases; mp/dp is above 300 °C in all the cases.

### Analysis and Physical Measurements

Mn, Fe, Co, Ni, Cu, Zn and Na were estimated by using Perkin-Elmer flame atomic absorption spectroscopy, model SOLAAR 32AA. Carbon, hydrogen and nitrogen analyses were carried out on a Perkin-Elmer CHN Elemental Analyzer, model 2400. Combustion temperature was 920°C and reduction temperature was 640°C.

Room temperature magnetic susceptibility measurements were carried out on a Faraday type balance (Cahn-electronic balance 75570) using catena-tetrathiocyanatocobalt(II) mercury(II), $[CoHg(SCN)_4]$ as standard. Experimental magnetic susceptibility ($\chi_M$) was corrected for diamagnetism using the procedure of Figgis and Lewis /23/. Electronic spectra (1500-200 nm) of the ligand and complexes were recorded on a CARY-2390 UV-Visible spectrophotometer in double distilled water using the technique of Lee *et al* /24/. IR spectra between 4000-400 $cm^{-1}$ were recorded on a JASCO FT/IR-5300 spectrophotometer in nujol mulls. The ESR spectra of Cu(II) complex of $NaH_2L$ was recorded on an X-band spectrometer model Varian-E-11 at liquid nitrogen temperature and room temperature in solid state using DPPH (αα-diphenyl, β-picryl hydrazyl) as <g> marker. The field setup was 3200 G and scan range was 2000G. Powder X-ray diffraction data were analysed by Ito's method /25/. Molar conductance of all the water-soluble complexes was measured at room temperature on a WTW conductivity bridge fitted with Philips magic eye.

### Antifungal activity

The fungi were isolated on PDA (250 g peeled potato, 20 g dextrose, 15 g agar powder and 1L distilled water) medium from their respective hosts collected from the experimental farm of Banaras Hindu University and incubated for one week at room temperature. The cultures were purified by single spore isolation on PDA slants.

Aqueous solutions of each metal salt, $MnCl_2{\cdot}4H_2O$, $FeSO_4{\cdot}7H_2O$, $CoSO_4{\cdot}7H_2O$, $NiSO_4{\cdot}7H_2O$, $CuSO_4{\cdot}5H_2O$, and $ZnSO_4{\cdot}7H_2O$, were prepared by dissolving the required amounts in double distilled water thereby making concentrations of 1000 ppm. Aqueous solutions of $Na_3H_2L$ was prepared by dissolving the required amount in double distilled water to make concentrationsof 1000 ppm. Also complexes of metal ions (Mn(II), Fe(II), Co(II), Ni(II), Cu(II) and Zn(II)) with the ligands $Na_3H_2L$ were dissolved in the required amount of double distilled water to make concentrations up to 1000 ppm. These solutions were diluted further by addition of double distilled water for preparing the required sets of metal mixture solutions, namely, A, $A_1$, $A_2$, B, $B_1$, $B_2$, 1, 2, 3, 4 etc.

Antifungal activity of the above metal complexes was studied on various fungi, namely, *Alternaria brassicicola*, *Alternaria brassicae*, *Alternaria solanae*, *Alternaria triticina*, *Curvularia species*, *Curvularia lunata*, *Helminthosporium* oryzae, *Collectotrichum capsici*, *Aspergillus niger*, *Aspergillus flavus*, and *Fusarium udum*, by using the spore germination technique /26/. For comparison, antifungal activity of the ligands was also determined on the same fungi.

The antifungal activity of the above water-soluble complexes, metal salts and ligands was also studied by poisoned food technique on the fungi, *Curvularia species*, *Curvularia lunala*, *Alternaria triticina*, *Alternaria brassicicola* and *Fusariam udum* /27/.

A drop of metal-complex solution was placed on a grease-free glass slide and 200-300 spores of the test fungi were placed with the help of a sterilized inoculation needle on the solution. The slides were then placed in a moist chamber and incubated at 25 ± 2°C, for 24 h. After incubation the spores were fixed and stained with lectophenol cotton blue, and spore germination was observed under a light microscope. Similar spore numbers of each fungus were mixed in sterilized distilled water, which served as control. For measurement of inhibition, the percentage germination was subtracted by a hundred to get percentage inhibition. All the experiments were conducted in triplicate. The data were subjected to students 't' test for statistical significance.

Mycellial growth of five fungi, with or without chemicals, was observed by taking dry weight of fungi grown in 150 ml conical flask. All the conical flasks were filled with 50 ml potato dextrose broth. Required amounts of the chemicals were then added to the broth to get the desired concentrations (250, 500 and 750 ppm) individually and in the mixture and dissolved and mixed thoroughly by shaking the flasks. After autoclaving for 15 min (at 121°C) the broth was allowed to cool down and a 5 mm disc of fungal mycelium was taken from the border of an actively growing fungal colony and inoculated into the broth. The flasks were incubated at 25 ± 2°C for one week. Potato dextrose broth without the chemicals served as control. After one week, the broth with the fungal colony was filtered on previously weighed filter papers and dried at 80°C for 24 h. The dry weight of the fungal colony was determined by deducting the weight of the filter paper from the total weight of filter paper and mycelium. All the experiments were conducted in triplicate. The data were subjected to student 't' test for statistical significance. Antifungal activity measured by these methods is presented in the Tables (8, 9, 10, 11, 12).

### Statistical analysis

The data recorded for different concentrations of metal-complex solutions were subjected to the following statistical analysis.

### Analysis of variance (ANOVA)

The analysis of variance was carried out separately for each fungus against all the compounds at various concentrations according to the procedure of Randomized Block Design Analysis /28/.

**Analysis of variance (ANOVA)**

| Source of variance | df. | S.S. | M.S. | $F_{cal}$ |
|---|---|---|---|---|
| Replication | (r-1) | RSS | RMS | RMS/ErMS |
| Concentration | (c-1) | CSS | CMS | CMS/ErMS |
| Error | (r-1) (c-1) | ErSS | ErMS | |
| Total | (rc -1) | TSS | | |

where:

r = number of replications; c = number of concentrations; d.f. = degree of freedom; S.S. = Sum of squares; M.S. = Mean sum of squares; $F_{Cal}$ = Calculated value of F

### RESULTS AND DISCUSSION

Analytical data of the complexes (Table 1) show that all the complexes of $Na_3H_2L$ with Mn(II), Fe(II), Co(II), Ni(II), Cu(II) and Zn(II), are colored, water soluble and have 1:2 M(II):ligand stoichiometry. The complexes are partially soluble in ethanol and insoluble in benzene, chloroform and acetone. Electrolytic conductance data indicate that all the complexes are ionic in nature.

Since $Na_3H_2L$ (Figure-1) has several alternative bonding sites, namely –OH, $-SO_3^-$ and –N=N-,

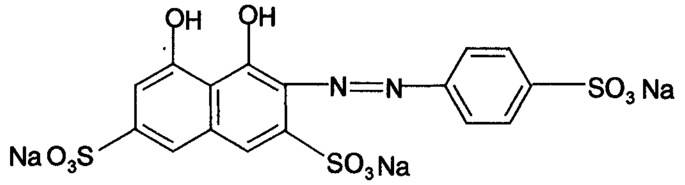

**Fig. 1:** 2-(4-sulphophenylazo)-1,8-dihydroxy 3,6 naphthalene disulphonic acid trisodium salt, $Na_3H_2L$

Conductance of aqueous solution (342 $\Omega^{-1}$ $cm^2$ $mol^{-1}$) suggest the following ionic species of the ligand $Na_3H_2L$.

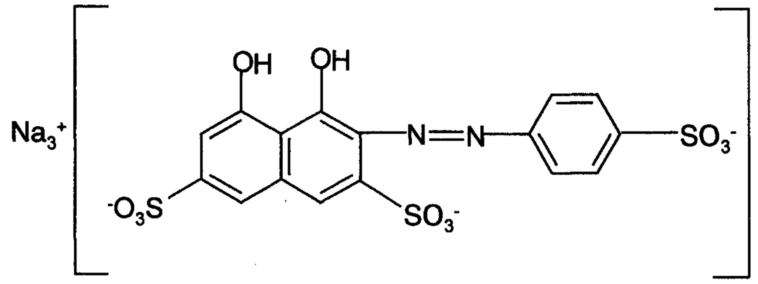

**Fig. 2 :** $Na_3^+(H_2L)^{3-}$

With bivalent metal ions the ligand may react as under,

$MSO_4 \cdot aH_2O + 2Na_3H_2L + 2NaOH \rightarrow Na_6[M(HL)_2(H_2O)_2] + Na2SO4 + aH2O$

(pH = 8-9)

where M = Fe, Co, Cu, Zn, a = 7 for Fe, Co, Ni, Zn and 5 for Cu
and

$MnCl_2 \cdot 4H_2O + 2Na_3H_2L + 2NaOH \rightarrow Na_6[M(HL)_2(H_2O)_2] + 2NaCl + aH2O$

(pH = 8-9)

Since $M^{2+}$ ions in aqueous solution are always hydrated as $M(H_2O)_6^{2+}$ and the analytical and the conductance (698-711 $\Omega^{-1}$ $cm^2$ $mol^{-1}$) data suggest 1:2 (M:L) complex formation, the probable metal-ligand bonding are shown in Figure 3 and Figure 4.

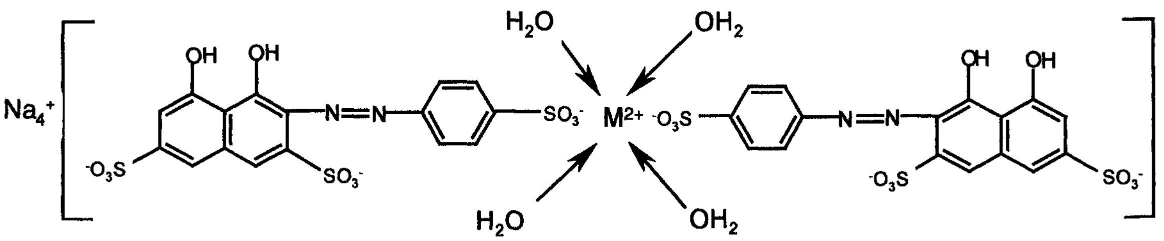

**Fig. 3:** Showing the possible of $M^{2+}$ in ionic bonded ligands

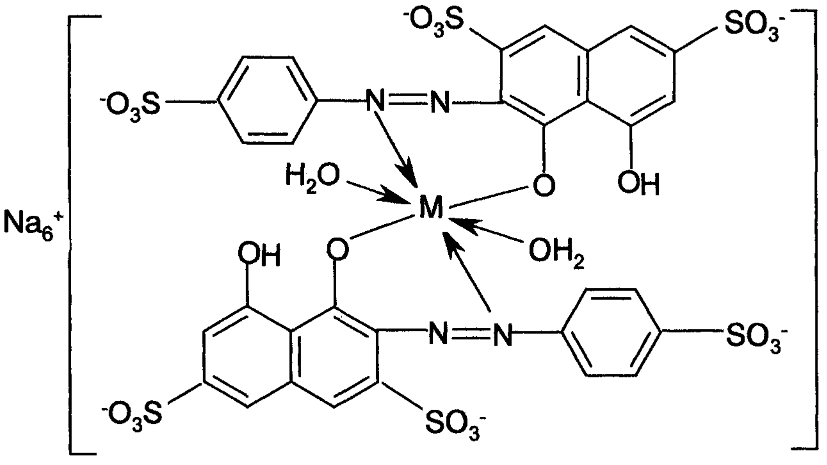

**Fig. 4:** Showing ionic structure of bis chelated $M^{2+}$ complex with the title ligand.

### IR Spectra

Aromatic α-hydroxy azo compounds, such as the present ligand, undergo fast intramolecular proton transfer between the enol-azo form and keto-hydrazone form. This equilibrium has been studied by a variety of spectroscopic techniques /8/.

Major peaks observed in the spectra of $Na_3H_2L$, at 3425, 1628 and 1508 $cm^{-1}$ (Table-2), were assigned to ν(OH), the ring vibrations and ν(N=N) respectively /29, 30/, while in the metal complexes peaks observed between 3500-3400, 1610-1580, 1400-1500 $cm^{-1}$ were identified and attributed to above group frequencies. A weak band observed at ca. 490-440 $cm^{-1}$, in the spectra of the complexes was assigned to the ν (M–N) vibration /31/.

A broad band centered between 3500-3400 $cm^{-1}$ in all the complexes arises due to ν(OH) of the coordinated $H_2O$. Besides, rocking ( ρr) and twisting (ρt) $H_2O$ modes between 912-823 $cm^{-1}$ and around 679-590 $cm^{-1}$, respectively, are observed in the complexes indicating coordinated water molecules /32/. Upon heating, above 150 °C, the colour of the complexes changed and the IR spectra showed disappearance of the $H_2O$ vibrations.

ν(S–O) in $Na_3H_2L$ at about 1020 $cm^{-1}$ remains unaffected in the complexes, indicating that the $-SO_3^-$ groups remain ionic even in complexes /33/.

**Table 2**

Tentative assignment of significant peaks ($cm^{-1}$) in the IR spectra of Mn(II), Fe(II), Co(II), Ni(II), Cu(II) and Zn(II) complexes with $Na_3H_2L$

| Name of compound/ peaks | ν(OH)/$H_2O$ | Aromatic ring vibrations | ν (N=N) | ν (S–O) | ν (M–N) |
|---|---|---|---|---|---|
| $Na_3H_2L$ | 3425 (s) | 1628(s) | 1508(m) | 1192(s)<br>636(m) | - |
| $Na_6[Mn(HL)_2(H_2O)_2]$ | 3449(s)<br>833(m)<br>630(w) | 1607(s) | 1500(m) | 1192(s)<br>638(m) | 488(w) |
| $Na_6[Fe(HL)_2(H_2O)_2]$ | 3418(s)<br>839(m)<br>590(w) | 1585(s) | 1460(m) | 1197(s)<br>626(m) | 447(w) |
| $Na_6[Co(HL)_2(H_2O)_2]$ | 3495(s)<br>912(m)<br>621(w) | 1591 | 1423(m) | 1191(s)<br>621(m) | 442(w) |
| $Na_6[Ni(HL)_2(H_2O)_2]$ | 3441(s)<br>833(m)<br>611(w) | 1608(s) | 1402(m) | 1190(s)<br>640(m) | 461(w) |
| $Na_6[Cu(HL)_2(H_2O)_2]$ | 3447(s)<br>840(m)<br>601(w) | 1607(s) | 1496(m) | 1195(s)<br>640(m) | 460(w) |
| $Na_6[Zn(HL)_2(H_2O)_2]$ | 3449(s)<br>823(m)<br>679(w) | 1610(s) | 1500(m) | 1190(s)<br>644(m) | 456(w) |

HL = $C_{16}H_8N_2O_{11}S_3$

### Electronic spectra

Absorption spectral data of the aqueous solutions of the complexes are reported in Table-3. These appear to be only charge transfer spectra with extinction coefficients, for the lowest band, around near $10^4$ $dm^3mol^{-1}cm^{-1}$. The lowest energy absorption band ($t_2g \rightarrow \pi^*$) /34/ is bathochromic shifted in the complexes /35/ (Table 3). The more intense band at higher energy is believed to be the spin allowed singlet-singlet transition while the weaker band at lower energy could be to the corresponding singlet-triplet transition mode particularly, allowed by the strong spin-orbit coupling in the ion /36/. The other probabilities, like low symmetry splitting of the metal orbitals and the presence of more than one entering ligand (each contributing one $\pi^*$ orbital), are excluded due to small extinction coefficient values /37/. Since the LMCT and MLCT have covered the entire visible region, the d-d electronic transition bands submerged under the CT bands, have not been identified.

**Table 3**

Tentative assignment of significant peaks in the electronic spectra of Mn(II), Fe(II), Co(II), Ni(II), Cu(II) and Zn(II) complexes with $Na_3H_2L$

| Complexes | $\lambda_{max}$ (nm) | $E_{max}$ x$10^4$ ($dm^3$ $mol^{-1}$ $cm^{-1}$) |
|---|---|---|
| $Na_6[Mn(HL)_2(H_2O)_2]$ | 226 | 50.92 |
| | 294 | 30.21 |
| | 362 | 15.61 |
| | 505 | 52.95 |
| $Na_6[Fe(HL)_2(H_2O)_2]$ | 224 | 51.66 |
| | 290 | 30.59 |
| | 366 | 15.63 |
| | 504 | 53.36 |
| $Na_6[Co(HL)_2(H_2O)_2]$ | 227 | 53.95 |
| | 301 | 30.93 |
| | 366 | 16.04 |
| | 512 | 53.95 |
| $Na_6[Ni(HL)_2(H_2O)_2]$ | 228 | 47.78 |
| | 298 | 30.72 |
| | 366 | 15.36 |
| | 510 | 53.24 |
| $Na_6[Cu(HL)_2(H_2O)_2]$ | 220 | 51.56 |
| | 288 | 30.25 |
| | 360 | 15.81 |
| | 508 | 53.28 |
| $Na_6[Zn(HL)_2(H_2O)_2]$ | 228 | 53.77 |
| | 306 | 31.02 |
| | 368 | 15.85 |
| | 515 | 53.77 |

HL = $C_{16}H_8N_2O_{11}S_3$

### Magnetic moments

The effective magnetic moments ($\mu_{eff}$) have been calculated from the room temperature magnetic susceptibility measurements ($\chi_g$), assuming Curie Law behaviour of the complexes and using literature values /38/ for diamagnetic corrections for the ions and also using directly measured corrections for the ligand /39/. The magnetic moments of Mn(II), Co(II), Fe(II), Ni(II), and Cu(II) complexes have been found in the range as expected for $O_h$ complexes (Table-1). The $\mu_{eff}$ value, 6.12 BM for Mn(II) complex lies well within the range for high spin $O_h$ complexes and 4.97 BM value for Fe(II) complex is diagnostic for $d^6$ system with some antiferromagnetic exchange interaction /40/. The $\mu_{eff}$ values for Co(II), Ni(II), Cu(II) have been found to be 4.59, 2.9 and 1.9 BM respectively, suggesting that all these ions have octahedral arrangements /41/ (Figure 5).

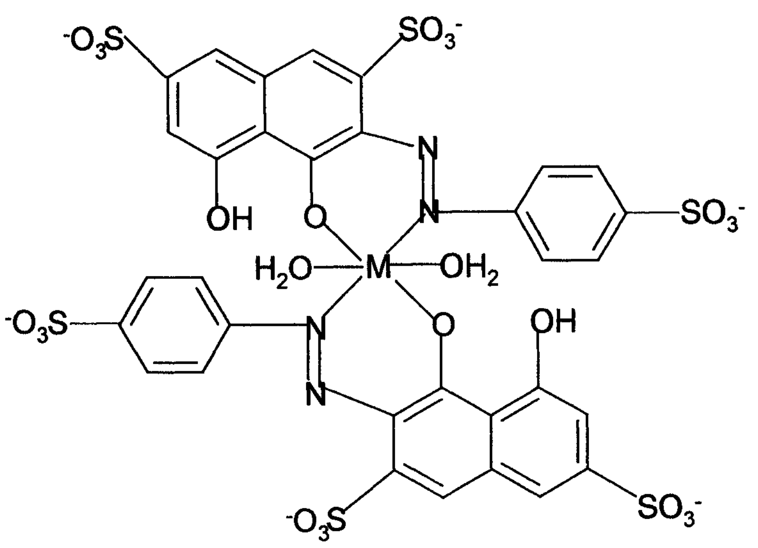

Mn(II), Fe(II), Co(II), Ni(II) & Zn(II) - Octahedral

Cu(II) - Distorted Octahedral

**Fig. 5:** Proposed anionic structure of Mn(II), Fe(II), Co(II), Ni(II), Cu(II) & Zn(II) complexes with $Na_3H_2L$.

### ESR Spectra

ESR spectra of metal complexes give useful information regarding the stereochemistry and nature of metal-ligand bonding /42/. Detailed ESR studies for metal-oxygen, metal-nitrogen and metal-sulphur bonding behaviour in the ligands have also been reported /43/.

ESR spectra of $Na_6[Cu(HL)_2(H_2O)_2]$ complex have been recorded at room temperature and liquid nitrogen temperature and the results are discussed below: Bonding parameters are included in Table 4 and the spectrum is illustrated in Figures 6 and 7 which was analyzed by the method of Knenbuhl /44/, Sands /45/ and German *et al.* /46/. Deviation of $g_{\parallel}$ value from that of free electron spin value (2.0023) and closeness of these values to those reported for distorted octahedral structure, suggest considerable distortion in the geometry of the complex /47, 48/.

Four lines have been observed in the LNT spectra, which remain unresolved at room temperature suggesting that a single copper ion is present in a molecule, or the complex is a monomer. G values are related by the expression, $G = \frac{g_{\parallel} - 2}{g_{\perp} - 2}$ and related to the exchange interaction between the copper centers, are greater than four, indicating negligible interaction between the two copper centers /49/.

**Table 4**

ESR parameters of Cu(II) complex with $Na_3H_2L$

| | $g_{\parallel}$ | $g_{\perp}$ | $g_{av}$ | G | $A_{\parallel} \times 10^{-4}$ $cm^{-1}$ | $A_{\perp} \times 10^{-4}$ $cm^{-1}$ | Aav. $\times 10^{-4}$ $cm^{-1}$ |
|---|---|---|---|---|---|---|---|
| At RT | 2.1311 | 2.0323 | 2.0652 | 4.0588 | 169.17 | 56.94 | 94.35 |
| At LNT | 2.1376 | 2.0336 | 2.0682 | 4.0952 | 174.68 | 61.73 | 99.39 |

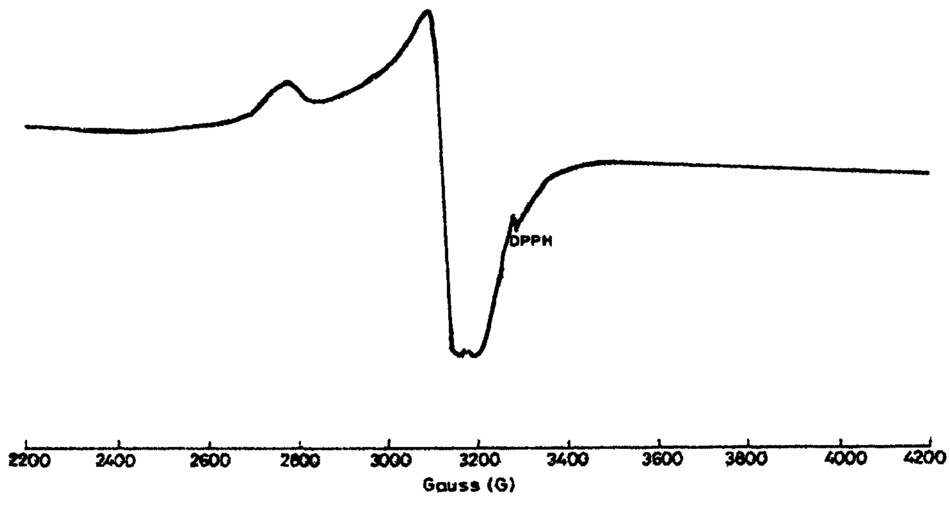

**Fig. 6:** Powder ESR spectrum of Cu(II) complex with $Na_3H_2L$ at RT

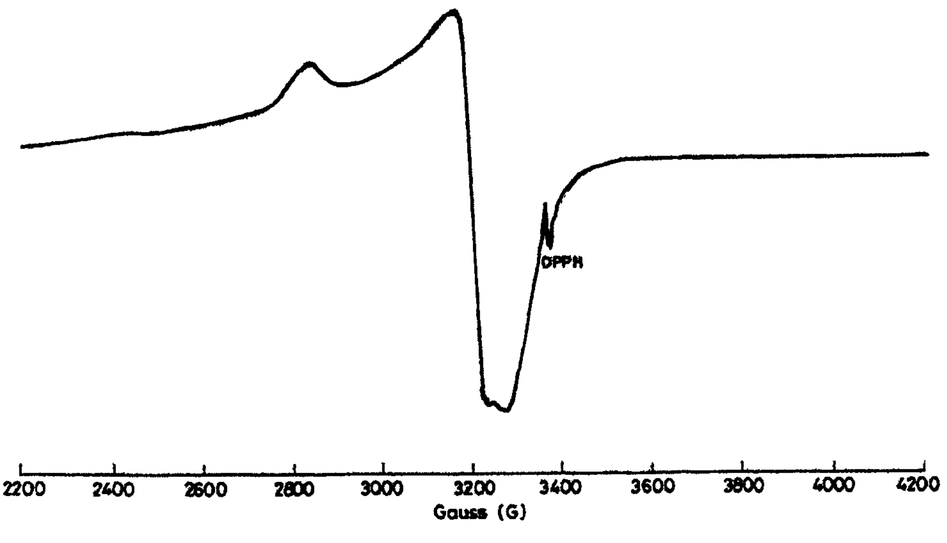

**Fig. 7:** Powder ESR spectrum of Cu(II) complex with $Na_3H_2L$ at LNT

### X-ray diffraction

The cell dimensions of the complex $Na_6[Fe(HL)_2(H_2O)_2]$, $Na_6[Fe(HL)_2(H_2O)_2]$ and $Na_6[Cu(HL)_2(H_2O)_2]$ are successfully calculated /25/ (Tables 5, 6, 7). The cell dimensions of $Na_6[Fe(HL)_2(H_2O)_2]$ are found to be: a = 20.0649, b = 19.1956, c = 15.1894, $\alpha$ = 95.0418, $\beta$ = 98.973 1, $\gamma$ = 126.8964 and V = 4497.7212 $Å^3$, $Na_6[Ni(HL)_2(H_2O)_2]$ are a = 17.6363, b = 14.9818, c = 12.0988, $\alpha$ = 87.2211, $\beta$ = 104.0606, $\gamma$ = 101.5108 and V = 3038.6613 $Å^3$ and of $Na_6[Cu(HL)_2(H_2O)_2]$ are found to be a = 16.1865, b = 20.5730, c = 14.1089, $\alpha$ = 138.1877, $\beta$ = 114.3296, $\gamma$ = 76.6370 and V = 2831.7911 $Å^3$. All these data agree with triclinic systems.

### Antifungal activity

Copper(II) sulphate and $Na_3H_2L$ tested individually as well as in mixture showed antifungal activity against all the fungi tested (Tables 8, 9, 10), though copper sulphate was much better than the $Na_3H_2L$, where spore germination of all the fungi was lower than $Na_3H_2L$ at 259 ppm concentration. Copper sulphate did not effect germination of *Curvularia lunata* significantly in its diluted form (Table 8) and similar results were obtained in case of $Na_3H_2L$ also (Table 9). Moreover, $Na_3H_2L$ in its diluted from $B_2$ (142 ppm) also did not effect spore germination of *Alternaria triticina* significantly. In all the other concentrations of the two compounds spore germination was affected significantly (Table 8, 9, 10). But the two compounds A (copper sulphate) and B (ligand) in their mixture (1:2) form showed more efficacy in inhibiting spore germination of *Curvularia lunata* and *A. triticina* at concentration 2(233 ppm) whereas at slightly higher concentration A(259 ppm) and B(285 ppm) the effect of individual compounds was not significant. The effect of mixed compounds and the individual compounds at the above concentrations showed almost similar results for the rest of the fungi (Tables 8, 9, 10).

When M(II) and ligands were mixed in ratio of 1:2, an excellent synergistic effect was noticed in the case of two of the tested fungi, viz., *Curvularia lunata* and *Alternaria triticina*. At concentration $A_1$ (129 ppm) and $B_1$(285 ppm), the effect of individual compounds was not significant against *Curvularia lunata* whereas when the two compounds were mixed even at the lowest concentration 4(58 ppm), there was significant reduction in spore germination. Similarly at $B_2$(142 ppm) spore germination of *Alternaria triticina* was not significantly reduced. But the two compounds in mixture inhibited spore germination significantly even at the lowest concentration 4(58 ppm). Similar results were obtained when 5 selected fungi were taken for their mycelial growth on potato dextrose broth supplemented with the chemicals. The spores which showed sensitivity against the chemicals also showed a similar trend in the production of mycelial dry weight. Out of the 5 tested fungi, *Alternaria triticina* showed maximum sensitivity when the chemicals were mixed, followed by *Alternaria brassicae* and *Fusarium udum* (Table 11).

The result of the present experiment showed the probable synergistic effect of the two compounds in the mixture. Such compounds may inhibit development of resistance since they have multisite action majority in comparison to widely used fungicides with single site of action. Further experimentation with these compounds in glasshouse and under field conditions is suggested for practical application of plant disease control.

The effect of ligand $Na_3H_2L$ was not very significant on the fungus *Altarnaria solanae* but it was very effective in case of Fe(II), Co(II), Ni(II), and Zn(II) complexes with $Na_3H_2L$ where the inhibition (percent)

**Table 5**

Powder X-ray diffraction data of the complex $Na_6[Fe(HL)_2(H_2O)_2]$

| Line | 2θ | Relative I | d | $1/d^2=Q_{obs}$ | $Q_{Cal}$ | Error in $Q_{obs}$ | h | k | l |
|---|---|---|---|---|---|---|---|---|---|
| 1 | 11.625 | 139 | 7.6059 | 0.01729 | 0.01729 | -0.00000 | $\bar{2}$ | 2 | 1 |
| 2 | 12.852 | 145 | 6.8826 | 0.02111 | 0.02159 | -0.00048 | $\bar{1}$ | 1 | 2 |
| 3 | 13.971 | 1000 | 6.3339 | 0.02493 | 0.02463 | +0.00030 | $\bar{2}$ | 3 | 0 |
| 4 | 14.892 | 138 | 5.9438 | 0.02831 | 0.02830 | +0.00001 | $\bar{1}$ | 3 | 0 |
| 5 | 15.828 | 129 | 5.5944 | 0.03195 | 0.03195 | 0.00000 | 2 | 1 | 0 |
| 6 | 16.879 | 188 | 5.2487 | 0.03630 | 0.03636 | -0.00006 | 0 | 2 | 2 |
| 7 | 17.774 | 485 | 4.9863 | 0.04022 | 0.04032 | -0.00010 | 0 | 3 | 0 |
| 8 | 19.117 | 597 | 4.6388 | 0.04647 | 0.04644 | +0.00003 | 0 | $\bar{3}$ | 2 |
| 9 | 21.876 | 143 | 4.0597 | 0.06068 | 0.06068 | 0.00000 | 1 | 3 | 0 |
| 10 | 22.947 | 464 | 3.8725 | 0.06668 | 0.06672 | -0.00004 | 4 | 0 | 0 |
| 11 | 23.816 | 635 | 3.7331 | 0.07176 | 0.07172 | +0.00004 | 0 | 3 | 2 |
| 12 | 24.362 | 409 | 3.6507 | 0.07573 | 0.07504 | +0.00069 | 0 | 0 | 4 |
| 13 | 25.893 | 538 | 3.4382 | 0.08459 | 0.08476 | -0.00017 | 3 | 0 | $\bar{4}$ |
| 14 | 28.184 | 428 | 3.1637 | 0.09991 | 0.10007 | -0.00016 | 3 | $\bar{5}$ | 3 |
| 15 | 29.504 | 267 | 3.0251 | 0.10927 | 0.10924 | +0.00003 | 4 | $\bar{5}$ | 3 |
| 16 | 30.110 | 114 | 2.9656 | 0.11370 | 0.11376 | -0.00006 | $\bar{5}$ | 2 | 4 |
| 17 | 30.612 | 145 | 2.9181 | 0.11744 | 0.11740 | +0.00004 | 1 | 2 | $\bar{5}$ |
| 18 | 32.342 | 967 | 2.7658 | 0.13072 | 0.13062 | +0.00010 | 4 | $\bar{6}$ | 3 |
| 19 | 33.320 | 646 | 2.6869 | 0.13852 | 0.13847 | +0.00005 | 6 | $\bar{6}$ | 2 |
| 20 | 34.226 | 245 | 2.6148 | 0.14626 | 0.14619 | +0.00007 | 7 | $\bar{2}$ | 0 |
| 21 | 35.662 | 119 | 2.5165 | 0.15802 | 0.15797 | +0.00005 | $\bar{3}$ | 5 | 4 |
| 22 | 36.691 | 108 | 2.4474 | 0.16695 | 0.16683 | +0.00012 | 1 | $\bar{6}$ | 4 |
| 23 | 37.632 | 93 | 2.3883 | 0.17532 | 0.17530 | +0.00002 | $\bar{7}$ | 7 | 2 |
| 24 | 38.236 | 134 | 2.3519 | 0.18078 | 0.18080 | -0.00002 | 4 | $\bar{8}$ | 0 |
| 25 | 39.259 | 275 | 2.2930 | 0.19019 | 0.19044 | -0.00025 | 2 | $\bar{6}$ | 5 |
| 26 | 39.848 | 109 | 2.2604 | 0.19752 | 0.19545 | +0.00207 | $\bar{8}$ | 7 | 2 |
| 27 | 40.633 | 145 | 2.2186 | 0.20316 | 0.20322 | -0.00006 | $\bar{7}$ | 4 | 5 |
| 28 | 42.707 | 112 | 2.1155 | 0.22345 | 0.22347 | -0.00002 | 3 | 4 | 2 |
| 29 | 44.916 | 92 | 2.0164 | 0.24595 | 0.24509 | +0.00086 | 8 | $\bar{2}$ | 2 |
| 30 | 45.621 | 102 | 1.9869 | 0.25331 | 0.25321 | +0.00010 | 5 | 3 | $\bar{5}$ |
| 31 | 47.112 | 118 | 1.9275 | 0.26916 | 0.26908 | +0.00008 | 6 | 3 | $\bar{3}$ |
| 32 | 51.195 | 100 | 1.7829 | 0.31459 | 0.31425 | +0.00034 | 8 | 1 | 0 |
| 33 | 53.326 | 223 | 1.7166 | 0.33936 | 0.33938 | -0.00002 | 10 | $\bar{5}$ | 3 |

**Table 5** (continued)

Powder X-ray diffraction data of the complex $Na_6[Fe(HL)_2(H_2O)_2]$

| Line | 2θ | Relative I | d | $1/d^2=Q_{obs}$ | $Q_{Cal}$ | Error in $Q_{obs}$ | h | k | l |
|---|---|---|---|---|---|---|---|---|---|
| 34 | 55.493 | 114 | 1.6546 | 0.36527 | 0.36521 | +0.00006 | 9 | $\bar{7}$ | 5 |
| 35 | 56.032 | 96 | 1.6399 | 0.37185 | 0.37188 | -0.00003 | $\bar{9}$ | 2 | 7 |
| 36 | 56.585 | 86 | 1.6252 | 0.37861 | 0.37862 | -0.00001 | $\bar{12}$ | 8 | 3 |
| 37 | 63.479 | 81 | 1.4643 | 0.46638 | 0.46641 | -0.00003 | $\bar{13}$ | 10 | 3 |
| 38 | 65.045 | 90 | 1.4328 | 0.48711 | 0.48711 | 0.00000 | 5 | 2 | $\bar{10}$ |
| 39 | 66.200 | 77 | 1.4105 | 0.50264 | 0.50257 | +0.00007 | 8 | $\bar{13}$ | 4 |
| 40 | 67.895 | 92 | 1.3794 | 0.52556 | 0.52566 | -0.00010 | 4 | $\bar{12}$ | 6 |
| 41 | 68.495 | 134 | 1.3688 | 0.53373 | 0.53373 | 0.00000 | $\bar{11}$ | 2 | 8 |
| 42 | 69.190 | 72 | 1.3567 | 0.54329 | 0.54330 | -0.00001 | 4 | $\bar{13}$ | 0 |

**Table 6**

Powder X-ray diffraction data of the complex $Na_6[Ni(HL)_2(H_2O)_2]$

| Line | 2θ | Relative I | d | $1/d^2=Q_{obs}$ | Q calc. | Error in Q | h | k | l |
|---|---|---|---|---|---|---|---|---|---|
| 1 | 12.089 | 60 | 7.3151 | 0.01869 | 0.01856 | +0.00013 | 0 | 2 | 0 |
| 2 | 15.091 | 38 | 5.8661 | 0.02906 | 0.02904 | +0.00002 | 0 | 0 | 2 |
| 3 | 17.840 | 52 | 4.9680 | 0.04052 | 0.04050 | +0.00002 | 1 | $\bar{1}$ | 2 |
| 4 | 18.714 | 44 | 4.7378 | 0.04455 | 0.04264<br>0.04543 | +0.00091<br>-0.00088 | 1<br>$\bar{1}$ | 1<br>3 | 2<br>1 |
| 5 | 22.719 | 35 | 3.9108 | 0.06538 | 0.06534<br>0.06540 | +0.00004<br>-0.00002 | 0<br>2 | 0<br>3 | 3<br>0 |
| 6 | 24.134 | 25 | 3.6847 | 0.07365 | 0.07374 | -0.00009 | 4 | 0 | 1 |
| 7 | 24.656 | 13 | 3.6078 | 0.07683 | 0.07634 | +0.00049 | $\bar{1}$ | 4 | 1 |
| 8 | 25.172 | 15 | 3.5350 | 0.08002 | 0.07971<br>0.08068 | +0.00031<br>-0.00066 | 4<br>$\bar{5}$ | $\bar{2}$<br>1 | 1<br>1 |
| 9 | 27.260 | 82 | 3.2688 | 0.09359 | 0.09359 | 0.00000 | 5 | 0 | $\bar{2}$ |
| 10 | 29.291 | 36 | 3.0466 | 0.10774 | 0.10710<br>0.10811 | +0.00064 -<br>0.00037 | 0<br>1 | 3<br>3 | $\bar{3}$<br>$\bar{3}$ |
| 11 | 30.408 | 37 | 2.9372 | 0.11591 | 0.11598 | -0.00007 | $\bar{4}$ | 3 | 3 |
| 12 | 31.316 | 16 | 2.8540 | 0.12277 | 0.12281 | -0.00004 | 4 | $\bar{4}$ | 1 |
| 13 | 31.978 | 15 | 2.7965 | 0.12787 | 0.12780<br>0.12788 | +0.00007 -<br>0.00001 | 6<br>5 | 0<br>2 | $\bar{2}$<br>$\bar{2}$ |
| 14 | 34.014 | 10 | 2.6336 | 0.14418 | 0.14410 | +0.00008 | 1 | $\bar{4}$ | 3 |

**Table 6** (continued)

Powder X-ray diffraction data of the complex $Na_6[Ni(HL)_2(H_2O)_2]$

| Line | 2θ | Relative I | d | $1/d^2{}_{=Qobs}$ | Q calc. | Error in Q | h | k | l |
|---|---|---|---|---|---|---|---|---|---|
| 15 | 34.938 | 23 | 2.5660 | 0.15188 | 0.15185 | +0.00003 | 2 | 4 | $\bar{3}$ |
| 16 | 36.524 | 30 | 2.4581 | 0.16550 | 0.16542 | +0.00008 | 5 | $\bar{3}$ | 0 |
| 17 | 37.626 | 14 | 2.3887 | 0.17526 | 0.17499 | +0.00027 | 3 | 3 | 3 |
| 18 | 38.326 | 90 | 2:3464 | 0.18160 | 0.18172 | -0.00012 | $\bar{2}$ | 6 | 2 |
| 19 | 38.815 | 28 | 2.3182 | 0.18608 | 0.18606<br>0.18607 | +0.00002<br>+0.00001 | 6<br>$\bar{6}$ | $\bar{4}$<br>4 | 1<br>3 |
| 20 | 41.345 | 14 | 2.1820 | 0.21003 | 0.21003 | +0.00000 | 4 | $\bar{1}$ | 4 |
| 21 | 43.106 | 21 | 2.0969 | 0.22743 | 0.22736 | +0.00007 | 0 | 7 | 0 |
| 22 | 45.052 | 13 | 2.0107 | 0.24735 | 0.24721 | +0.00014 | 8 | $\bar{2}$ | 1 |
| 23 | 45.844 | 222 | 1.9778 | 0.25564 | 0.25574 | -0.00010 | 0 | 4 | 5 |
| 24 | 46.405 | 1000 | 1.9552 | 0.26159 | 0.26158 | +0.00001 | 6 | 4 | $\bar{3}$ |
| 25 | 46.998 | 17 | 1.9319 | 0.26794 | 0.26763<br>0.26818 | +0.00031<br>-0.00024 | $\bar{1}$<br>$\bar{8}$ | 6<br>5 | 4<br>1 |
| 26 | 48.504 | 48 | 1.8754 | 0.28432 | 0.28505 | -0.00073 | 4 | $\bar{1}$ | 5 |
| 27 | 51.612 | 13 | 1.7695 | 0.31937 | 0.31939 | -0.00002 | $\bar{7}$ | 7 | 2 |
| 28 | 52.940 | 20 | 1.7282 | 0.33482 | 0.33484 | -0.00002 | $\bar{10}$ | 2 | 3 |
| 29 | 54.695 | 91 | 1.6768 | 0.35566 | 0.35563<br>0.35569 | +0.00003<br>-0.00003 | 8<br>2 | 2<br>8 | 5<br>$\bar{2}$ |
| 30 | 55.692 | 12 | 1.6508 | 0.36695 | 0.36667 | +0.00028 | 3 | 8 | 0 |
| 31 | 56.990 | 9 | 1.6146 | 0.38359 | 0.38321 | +0.00038 | 5 | 4 | $\bar{6}$ |
| 32 | 64.179 | 12 | 1.4500 | 0.47562 | 0.47565 | -0.00003 | 4 | 4 | 6 |

**Table 7**

Powder X-ray diffraction data of the complexes $Na_6[Cu(HL)_2(H_2O)_2]$

| Line | 2θ | Relative I | d | $1/d^2$ = $Q_{obs}$ | Q calc. | Error | h | k | l |
|---|---|---|---|---|---|---|---|---|---|
| 1 | 10.931 | 254 | 8.0872 | 0.01529 | 0.01535 | -0.00006 | 1 | $\bar{1}$ | 1 |
| 2 | 12.945 | 496 | 6.8332 | 0.02142 | 0.02157 | -0.00015 | 2 | $\bar{1}$ | 0 |
| 3 | 18.220 | 179 | 4.8653 | 0.04225 | 0.04225 | 0.00000 | 3 | 2 | $\bar{1}$ |
| 4 | 20.303 | 1000 | 4.3705 | 0.05235 | 0.05236 | +0.00099 | 0 | 0 | 2 |
| 5 | 22.098 | 173 | 4.0914 | 0.05974 | 0.05971 | +0.00003 | 3 | 0 | $\bar{2}$ |
| | | | | | 0.05975 | -0.00001 | 2 | $\bar{3}$ | 0 |
| 6 | 24.974 | 622 | 3.5626 | 0.07879 | 0.07933 | -0.00054 | 3 | $\bar{3}$ | 0 |
| 7 | 26.161 | 453 | 3.4036 | 0.08632 | 0.08629 | +0.00003 | 3 | 4 | $\bar{4}$ |
| | | | | | 0.08640 | -0.00008 | 0 | 4 | 0 |
| 8 | 29.106 | 321 | 3.0655 | 0.10641 | 0.10636 | +0.00005 | 4 | 2 | 0 |
| 9 | 30.526 | 661 | 2.9261 | 0.11679 | 0.11675 | +0.00004 | 5 | 0 | 0 |
| 10 | 32.133 | 255 | 2.7833 | 0.12909 | 0.12907 | +0.00002 | 3 | 0 | 2 |
| 11 | 37.219 | 245 | 2.4138 | 0.17163 | 0.17156 | +0.00007 | 2 | 8 | $\bar{3}$ |
| 12 | 39.729 | 197 | 2.2670 | 0.19458 | 0.19440 | +0.00018 | 0 | 6 | 0 |
| | | | | | 0.19466 | -0.00008 | 3 | $\bar{7}$ | 2 |
| 13 | 41.302 | 744 | 2.1841 | 0.20963 | 0.20953 | +0.00010 | $\bar{6}$ | 3 | 1 |
| | | | | | 0.20975 | -0.00012 | 0 | 5 | 1 |
| 14 | 45.315 | 161 | 1.9996 | 0.25010 | 0.25014 | -0.00004 | 6 | 8 | $\bar{4}$ |
| 15 | 46.126 | 365 | 1.9663 | 0.25864 | 0.25866 | -0.00002 | 1 | 4 | 2 |
| 16 | 51.858 | 246 | 1.7680 | 0.31992 | 0.31988 | +0.00004 | 7 | $\bar{5}$ | 0 |
| | | | | | 0.32001 | -0.00009 | 6 | 2 | $\bar{6}$ |
| 17 | 53.689 | 167 | 1.7058 | 0.34367 | 0.34370 | -0.00003 | 4 | 1 | $\bar{6}$ |
| 1 | 63.662 | 173 | 1.4814 | 0.45568 | 0.45570 | -0.00002 | 10 | 6 | $\bar{7}$ |
| 19 | 66.041 | 138 | 1.4135 | 0.50050 | 0.50059 | -0.00009 | 11 | 4 | $\bar{6}$ |

**Table 8**

Effect of copper sulphate on spore germination of some fungi

| Fungus / Treatment | Host | Control | A | $A_1$ | $A_2$ |
|---|---|---|---|---|---|
| *Aspergillus niger* | *Saprophyte* | 66.33 | 3.17** | 8.33** | 15.83** |
| *Aspergillus flavus* | *Saprophyte* | 80.33 | 2.83** | 22.5** | 41.67** |
| *Helminthosporium oryzae* | *Oxyza sativa* | 96.72 | 2.97** | 5.67** | 6.59** |
| *Curvularia sp.* | *Brassica campestris* | 96.33 | 3.63** | 4.95** | 18.4** |
| *Curvularia lunata* | *O. sativa* | 97.33 | 68.89** | 82.73 | 87.54 |
| *Colletorichum capsici* | *Capsicum annuum* | 63.57 | 0.63** | 2.87** | 4.95** |
| *Alternaria brassicae* | *B. campestris var. capitata* | 95.53 | 2.00** | 418** | 17.37** |
| *Alternaria triticina* | *Triticum aestivum* | 99.27 | 18.03** | 24.43** | 70.15** |
| *Alternaria brassicicola* | *B. campestris* | 92.63 | 7.56** | 11.24** | 27.26** |
| *Fusarium udum* | *Cajanus cajan* | 98.63 | 2.53** | 11.25** | 18.86** |

Row data with ** are significant at $p \geq 0.01$

**Table 9**

Effect of $Na_3H_2L$ on spore germination of some fungi

| Fungus / Treatment | Host | Control | B | $B_1$ | $B_2$ |
|---|---|---|---|---|---|
| *Aspergillus niger* | *Saprophyte* | 66.33 | 9.67** | 12.45** | 19.05** |
| *Aspergillus flavus* | *Saprophyte* | 80.33 | 7.67** | 24.83** | 47.00** |
| *Helminthosporium oryzae* | *Oxyza sativa* | 96.72 | 6.57** | 9.33** | 52.17** |
| *Curvularia species* | *Brassica campestris* | 96.33 | 13.21** | 24.86** | 36.72** |
| *Curvularia lunata* | *O. sativa* | 97.33 | 73.00** | 82.73 | 87.54 |
| *Colletorichum capsici* | *Capsicum annuum* | 63.57 | 5.83** | 8.89** | 12.70** |
| *Alternaria brassicae* | *B. campestris var. capitata* | 95.53 | 14.14** | 21.69** | 33.49** |
| *Alternaria triticina* | *Triticum aestivum* | 99.27 | 49.39** | 71.83** | 87.87** |
| *Alternaria brassicicola* | *B. campestris* | 92.63 | 24.89** | 34.4** | 41.87** |
| *Fusarium udum* | *Cajanus cajan* | 98.63 | 9.78** | 15.22** | 41.25** |

Row data with ** are significant at $p \geq 0.01$

**Table 10**

Effect of $Na_6[Cu(HL)_2(H_2O)_2]$on spore germination of some fungi

| Fungus / Treatment | Host | Control | 1 | 2 | 3 | 4 |
|---|---|---|---|---|---|---|
| *Aspergillus niger* | *Saprophyte* | 92.75 | 8.36** | 10.17** | 20.16** | 25.35** |
| *Aspergillus flavus* | *Saprophyte* | 94.32 | 6.17** | 13.86** | 26.06** | 29.20** |
| *Helminthosporium oryzae* | *Oxyza sativa* | 84.78 | 3.44** | 4.93** | 8.58** | 17.97** |
| *Curvularia species* | *Brassica campestris* | 86.39 | 1.18** | 3.37** | 10.74** | 20.59** |
| *Curvularia lunata* | *O. sativa* | 91.39 | 3.94** | 11.19** | 23.32** | 37.23** |
| *Colletorichum capsici* | *Capsicum annuum* | 63.57 | 1.22** | 2.22* | 4.41** | 8.17** |
| *Alternaria brassicae* | *B. campestris var. capitata* | 92.47 | 2.18** | 4.92** | 12.53** | 20.16** |
| *Alternaria triticina* | *Triticum aestivum* | 93.32 | 0.60** | 10.72** | 27.31** | 63.11** |
| *Alternaria brassicicola* | *B. campestris* | 92.63 | 11.52** | 21.58** | 29.95** | 37.76** |
| *Fusarium udum* | *Cajanus cajan* | 98.63 | 4.72** | 9.54** | 17.34** | 24.08** |

Row data with ** are significant at $p \geq 0.01$

* Strength of solutions. (in ppm) 1-466.61
2-233.305
4-058.326
3-116.652

**Table 11**

Effect of $Na_3H_2L$ and copper sulphate on mycelial growth of some fungi.

| Treatment (ppm) | *Curvlaria lunata* | *Fusarium udum* | *Alternaria brassicae* | *Alteraria triticina* | *Curvularia species* |
|---|---|---|---|---|---|
| Control | 0.2301 | 0.2195 | 0.2180 | 0.2376 | 0.2487 |
| $CuSO_4 \cdot 5H_2O$ (250) | 0.1877 | 0.1498** | 0.1336** | 0.1438** | 0.2103 |
| $CuSO_4 \cdot 5H_2O$ (500) | 0.1318** | 0.1138** | 0.1090** | 0.1140** | 0.1863** |
| $CuSO_4 \cdot 5H_2O$ (750) | 0.1029** | 0.1013** | 0.1130** | 0.1020** | 0.1121** |
| $Na_3H_2L$ (250) | 0.1768** | 0.1588** | 0.2290** | 0.2130** | 0.1667** |
| $Na_3H_2L$ (500) | 0.1478** | 0.1289** | 0.2210** | 0.1860** | 0.1361** |
| $Na_3H_2L$ (750) | 0.0998** | 0.1075** | 0.2100** | 0.1470** | 0.0863** |
| $CuSO_4 \cdot 5H_2O$+ $Na_3H_2L$ (250) | 0.1431** | 0.1186** | 0.1010** | 0.1240** | 0.1422** |
| $CuSO_4 \cdot 5H_2O$ +$Na_3H_2L$ (500) | 0.1237** | 0.0981** | 0.0826** | 0.0930** | 0.1082** |
| $CuSO_4 \cdot 5H_2O$+ $Na_3H_2L$ (750) | 0.0857** | 0.0581** | 0.0496** | 0.0230** | 0.0637** |

Column data with ** are significant at $p \geq 0.01$

**Table 12**

Effect of $Na_3H_2L$ and complexes of $Na_3H_2L$, $Na_6[M(HL)_2(H_2O)_2]$on spore germination of some fungi (% inhibition)

| Fungus/ Treatment | Host | Control | $Na_3H_2L$ | | $Na_6[Mn(HL)_2(H_2O)_2]$ | | $Na_6[Fe(HL)_2(H_2O)_2]$ | | $Na_6[Co(HL)_2(H_2O)_2]$ | | $Na_6[Ni(HL)_2(H_2O)_2]$ | | $Na_6[Cu(HL)_2(H_2O)_2]$ | | $Na_6[Zn(HL)_2(H_2O)_2]$ | |
|---|---|---|---|---|---|---|---|---|---|---|---|---|---|---|---|---|
| | | | 1 | 2 | 1 | 2 | 1 | 2 | 1 | 2 | 1 | 2 | 1 | 2 | 1 | 2 |
| *Alternaria brassicae* | *B.Campestris Var.capitiata* | 7.34 | 26.0 | 4.5 | 15.0 | 3.4 | 29.5 | 8.7 | 28.0 | 5.8 | 32.3 | 1.3 | 86.6 | 78.2 | 21.0 | 6.7 |
| *Alternaria brassicicola* | *B.Campestris* | 1.67 | 99.5 | 92.5 | 49.8 | 23.4 | 75.0 | 40.1 | 91.5 | 76.7 | 100.0 | 96.9 | 77.9 | 55.4 | 79.0 | 88.2 |
| *Alternaria solanae* | *Solenum tuberosum* | 10.0 | 52 | 24.5 | 48.0 | 36.5 | 71.2 | 46.7 | 87.2 | 68.7 | 100.0 | 98.8 | 59.9 | 39.5 | 94.0 | 81.4 |
| *Curvularia lunata* | *O. sativa* | 32.18 | 99.05 | 92.5 | 99.5 | 85.6 | 99.1 | 37.7 | 99.1 | 92.1 | 99.4 | 90.7 | 98.7 | 93.7 | 96.3 | 84.9 |
| *Helminthosporium orazae* | *Oxyza sativa* | 1.17 | 100.0 | 98.4 | 26.5 | 4.5 | 35.3 | 4.5 | 22.5 | 9.8 | 84.6 | 3.4 | 91.3 | 77.9 | 98.9 | 91.7 |
| *Curvularia species* | *Brassica Campestris* | 3.67 | 98.77 | 86.17 | 99.2 | 85.5 | 94.5 | 66.7 | 99.5 | 97.3 | 100.0 | 98.1 | 100.0 | 98.8 | 81.5 | 78.3 |
| *Colletotrichum capsici* | *Capsicum annum* | 36.43 | 99.7 | 94.13 | 72.5 | 58.4 | 100.0 | 27.5 | 100.0 | 96.8 | 100.0 | 92.2 | 100.0 | 98.8 | 74.6 | 58.2 |
| *Aspergillus niger* | *Saprophyte* | 33.61 | 98.8 | 90.3 | 83.3 | 78.5 | 99.2 | 85.5 | 99.1 | 98.6 | 98.6 | 87.8 | 99.5 | 94.7 | 98.2 | 88.1 |
| *Aspergillus flavus* | *Saprophyte* | 19.67 | 99.7 | 92.27 | 56.7 | 27.4 | 99.5 | 80.4 | 94.6 | 80.5 | 75.4 | 55.3 | 99.4 | 95.8 | 85.4 | 82.7 |
| *Fusarium udum* | *Cajanus cajan* | 1.37 | 99.37 | 90.2 | 98.6 | 88.3 | 94.4 | 78.5 | 90.3 | 76.6 | 97.2 | 78.8 | 100.0 | 95.4 | 96.2 | 87.7 |

Strength of Solutions (in ppm)

1. 1000
2. 500

was 71, 87, 100 and 94, showing excellent synergetic effect (Table 12). In case of ***Collectrotrichun capscici, Aspergillus niger*** and ***Fusarium udum*** the effects of ligand, $Na_3H_2L$, as well as Mn(II), Fe(II), Co(II), Ni(II), Cu(II) and Zn(II) complexes with $Na_3H_2L$, were very significant showing inhibition up-to 100 percent in many of these cases.

From an overall study of the effect of the different metal salts, ligand $Na_3H_2L$, and its Mn(II), Fe(II), Co(II), Ni(II), Cu(II), and Zn(II) complexes, it is inferred that the metal complexes are better fungicidal agents than their parent ligands. Again in certain cases the complexes (metal salts + ligands) are more effective i.e., show more fungi-toxicity in comparison to the individual metal salts or the ligands, thereby indicating a good synergistic effect or the effect of complexation in solution.

For practical utility of these compounds, the inhibiting capacity of the complexes was compared with commercially available fungicides, e.g. dithane-M-45 (a broad fungicide) which is used in the inhibition of spore germination in the 0.1 – 0.2% in the field condition limit for many fungi. It was found that in the case of $Na_6[Ni(HL)_2(H_2O)_2]$ against *Curvularia species, Colletotricum capsici, Alternaria brassicicola* and *Alternaria solanae* and $Na_6[Cu(HL)_2(H_2O)_2]$ against *Curvularia species, Colletotricum capsici, Fusarium udum*, etc., the effect of the complexes was found to be better than that of commercially available fungicides e. g. dithane M-45.

This observation is quite significant and opens up a new field of research where metal complexes of organic molecules are better fungi-toxic than commercial products, showing greater possibility of applicability of these complexes under field conditions. A detailed mechanistic study may be desirable in future.

### ACKNOWLEDGEMENTS

One of the authors, Mr. Gajanan Pandey, is grateful to CSIR New Delhi for financial support as SRF. The authors also thank Prof. U. P. Singh, department of Mycology and Plant Pathology, Institute of Agricultural Sciences, Banaras Hindu University, Varanasi for helping antifungal activity measurement in his laboratory.

### REFERENCES

1. G. Banejee, *Analyt. Chim. Acta.*, **13**, 409 (1955).
2. A. K. Dey, S. N. Sinha, S. P. Sahgal, and K. N. Munshi, *Proc. $5^{th}$ Rare Earth Research Conference*, Ames, Iowa 1965.
3. A. K. Bhattacharjee and M. K. Mahanti, *Indian J. Chem.*, **20A**, 427 (1981).
4. N. K Dutta, *Chim. Analyst*, **42**, 562 (1960).
5. U. C. Maiwal and K. Srinivasulu, *J. Ind. Chem. Soc.*, **60**, 1086 (1983).
6. G. N. Ledesma, M. Gonzalez-Sierra and G. M. Escandersma, *Polyhderon*, **17**, 1517 (1998).
7. K. Berger, In: *Biocoordination Chemistry, Coordination Equilibria in Biologically Active Systems*, Ellis Horwood: New York, 1990.

8. G. A. Ibanez, C. Oliveri and G. M. Escander, *J. Chem. Soc. Faraday Trans.*, **93**, 545 (1997).
9. M. S. Rizk, M. A. Khaled-Abdel-Ghani and M. H. El-Kottamy, *Egypt. J. Chem.*, **36**, 199 (1993).
10. W. E. Antholine, J. M. Knight and D. H. Petering, *Inorg. Chem,* **16**, 569 (1977).
11. D. H. Jones, R. Slack, S. Squires and K. R. H. Wooldrige, *J. Med. Chem.*, 8, 676 (1965).
12. D. W. Klayman, J. P. Sconill, J. F. Bafosevich and J. Bruce, *J. Med. Chem.*, **26**, 35 (1983).
13. K. C. Agrawal and A. O. Santoelli, *Prog. Med. Chem.*, **15**, 349 (1978).
14. S. Dutta, P. Basu and A. Chakravorty, *Inorg. Chem.*, **32**, 5343 (1993), *Inorg. Chem,* **30**, 4031 (1991).
15. M. Ghisletta, H. P. Jalett, J. Gerfin, V. Gramlich and K. Hegetschweiler, *Helv. Chim, Acta,* **75**, 2233 (1992).
16. W. H. Ojala, E. A. Sudbeck, L. Khanh-Lu, T. I. Richardson, R. E. Lovrien and W. B. Gleason, *J. Am. Chem. Soc.*, **118**, 2231 (1996).
17. A. Balasubrahmanyan, V. K. Baranwal, M. L. Lodah, A. Varma and H. C. Kapoor, *Plant Science (Limerick)*, **154(1)**, 13 (2000).
18. D. Peciulyte and A. Lugauskas, *Proceedings of the International Conference: Development of Environmentally Friendly Plant Protection in Baltic Region, Tartu, Estonic*, September 28-29, (2000), [ed. by L. Metspalu and S.Mitt].
19. M. J. M. Campbell, *Coord. Chem. Rev.*, **15**, 279 (1975).
20. J. D. Leach, R. H. Garber and W. H. Lange, *Plant Disease Reptr. Suppl.*, **259**, 213 (1959).
21. G. Cik, A. Bujdakova and F. Sersen, *Chemosphere,* **44(3)**, 313 (2001).
22. C. Sarzanini, O. Abollino, M. C. Bruzzoniti and E. Mentasti, *Journal of Chromatography,* **804A**, 241 (1998).
23. B. N. Figgis and J. Lewis, In: *Modern Coordination Chemistry*, Ed. Lewis, J.; Wilkins, R. C. Interscience: New York, 1964, p 403.
24. R. H. Lee, G. Griswold and J. Kleinbery, *Inorg. Chem,* **3**, 1278 (1960).
25. L. V. Azaroff and M. J. Buerger, In: *The Powder Method in X-ray Crystallography.* McGraw-Hill Book Company: New York, 1958, p 105.
26. U. P. Singh, V. N. Pandey, K. G. Wagnar and K.P. Singh, *Can. J. Bot.*, **68**, 1354 (1990).
27. M. Tzatzarakis, M. Ksatsakis, A. Liakau and D.L. Vakalaunakie, *Journal of Environmental Science and Health,* Part B, *Pesticides Food Contaminants and Agricultural Wastes*, **35(4)**, 527 (2000).
28. V. G. Panse and P. V. Sukhatme, In: *Statistical Method for Agricultural Workers.* C. G. Raghava Kurup, Ed., ICAR, New Delhi, 1967, pp 152-159.
29. K. Krishnankuttey and J. Michel, *J. Coord. Chem.*, **22**, 327 (1991).
30. M. S. Sastry, U. P. Singh, R. Ghose and A. K. Ghose, *Synt. React. Inorg. Met-Org. Chem.*, **21**, 78 (1991).
31. B. T. Khan, K. V. Kumar and K. M. Mohan, *Ind. J. Chem.*, **31A**, 28 (1992).
32. K. Nakamoto, In: *Infrared and Raman Spectra of Inorganic and Coordination Compounds* Part B, 1997, p 54.
33. I. P. Saraswat, C. L. Sharma and A. Sharma, *J. Ind. Chem. Soc.*, **30**, 119 (1983).
34. B. K. Ghosh and A. Chakravorty, *Coord. Chem. Rev.*, **95**, 239 (1989).
35. R. A. Krause and A. Krause, *Inorg. Chem.*, **23**, 2195 (1984).

36. a. E. M. Kober and T. Meyer, *J. Inorg. Chem.*, **21**, 3967 (1982).
b. S. Decurtines, F. Felix, J. Ferguson, H. Gudel and H. U. Ludi, *J. Am. Chem. Soc.*, **102**, 4102 (1980).
37. T. K. Misra, D. Das, C. Sinha, P. Ghosh and C. K. Pal, *Inorg. Chem.*, **37**, 1672 (1998).
38. B. N. Figgis and J. Lewis, In: *Modern Coordination Chemistry* Lewis, J.; Wilkins, R. G. Eds., Ch. 6., Interscience Publishers; New York, 1960.
39. C. A. L. Becker, *J. Coord. Chem.*, **28**, 147 (1993).
40. R. L. Dutta and A. Syamal, In: *Element of Magnet Chemistry,* East West Press: Delhi, 1993.
41. B. N. Figgis and J. Lewis, In: *Progress in Inorganic Chemistry* F. A. Cotton; Interscience: New York, 1964, Vol. 6.
42. D. W. Smith, *J. Chem. Soc.* (A), **2**, 3108 (1970).
43. H. N. Chary, K. V. R. Chary, B. A. Sastry, and G. Ponticeley, *Ind. J. Pure & App. Phys.,* **15**, 550 (1960).
44. F. K. Kneubuhl, *J. Chem. Phys.*, **33**, 1074 (1960).
45. R. H. Sands, *Phys. Rev.*, **99**, 1222 (1955).
46. H. R. Germsan and J. D. Swalen *J. Chem. Phys.,* **56**, 3221 (1962).
47. D. Kivelson and R. Neiman, *J. Chem. Phys.,* **35**, 149 (1961).
48. B. J. Hathways and A. A. G. Tomlinson, *Coord. Chem. Rev.,* **5**, 1 (1970).
49. B. J. Hathway and D. E. Billing, *Coord.Cchem. Rev.*, **6**, 143 (1970).

